# SEC5 is involved in M2 polarization of macrophages *via* the STAT6 pathway, and its dysfunction in decidual macrophages is associated with recurrent spontaneous abortion

**DOI:** 10.3389/fcell.2022.891748

**Published:** 2022-10-14

**Authors:** Long Yang, Xuan Zhang, Yan Gu, Yan Shi, Long-Bin Wang, Jia-Xin Shi, Xing-Xing Zhen, Ya-Wei Xin, Wen-Wen Gu, Jian Wang

**Affiliations:** ^1^ NHC Key Lab of Reproduction Regulation (Shanghai Institute for Biomedical and Pharmaceutical Technologies), Fudan University, Shanghai, China; ^2^ The Second Hospital of Tianjin Medical University, Tianjin, China

**Keywords:** SEC5, STAT6, macrophage polarization, maternal-fetal interface, recurrent spontaneous abortion

## Abstract

Decidual macrophages (dMϕs) play critical roles in the establishment of microhomeostasis at the maternal-fetal interface during pregnancy. Impaired macrophage polarization during early pregnancy is associated with recurrent spontaneous abortion (RSA). In the present study, the SEC5 expression level was found to be significantly decreased in primary dMϕs of patients with RSA, and downregulation of SEC5 expression inhibited M2 polarization and STAT6 phosphorylation, whereas SEC5 overexpression in the Mϕs promoted M2 polarization and STAT6 phosphorylation *in vitro*. We subsequently found that SEC5 interacted with STAT6 in THP-1-derived Mϕs. The abundance of phosphorylated STAT6 (pSTAT6) protein was obviously increased, with a predominant distribution in the nucleus, after M2 polarization of Mϕs, and SEC5 protein was colocalized with pSTAT6. Moreover, a significantly reduced pSTAT6 expression level was observed in the dMϕs of patients with RSA. M2 polarization of Mϕs showed a stimulatory effect on the proliferation and invasion of human extravillous trophoblasts (EVTs) *in vitro*, and downregulation of SEC5 expression in Mϕs effectively reversed this effect. In a mouse model of LPS-induced early pregnancy loss, the uterine SEC5 expression level and the number of M2-Mϕs at the maternal-fetal interface were significantly reduced. More interestingly, heterozygous SEC5-deficient (SEC5^−/+^) pregnant mice were more sensitive to LPS-induced pregnancy loss. Taken together, these data indicate that SEC5 participates in the regulation of M2 polarization of Mϕs by interacting with STAT6 and that decreased SEC5 expression inhibits the M2 polarization of dMϕs and results in early pregnancy loss by interfering with the physical activities of EVTs and immunotolerance at the maternal-fetal interface.

## Background

Pregnancy is considered a unique immunological paradox because the maternal immune system is challenged by the semiallogeneic fetus during pregnancy, leading to a series of systemic and local immune responses (Erlebacher, 2013; Ander et al., 2019). Local immunologic homeostasis at the maternal-fetal interface must be strictly regulated not only to tolerate the presence of the fetus but also to protect the mother and the fetus against pathogens, leading to reproductive success (Guo et al., 2021). Failure to maintain such homeostasis may result in pregnancy complications, including recurrent spontaneous abortion (RSA, also called recurrent pregnancy loss, RPL, or recurrent miscarriage, RM) (Arck and Hecher, 2013; Lindau et al., 2018; Wang et al., 2019).

RSA is usually defined as the loss of two or more pregnancies before 20 weeks of gestation (Carp, 2019) and can cause serious physical and mental harm to women who suffer from it. Although the etiology of approximately 50% of RSA cases remains unknown, immune dysfunction is undoubtedly a main cause of unexplained RSA (URSA) (Triggianese et al., 2016; Guo et al., 2021). Pregnancy loss related to immune impairment is usually associated with disturbed maternal immune tolerance accompanied by systemic and local increases in proinflammatory cytokines and activated immune responses (Li et al., 2019). However, the specific mechanisms underlying deregulation of the local immune microenvironment remain unclear.

Decidual macrophages (dMϕs) are the second largest population (approximately 10%–20%) of decidual immune cells (DICs) and participate in embryo implantation, decidual vascular remodeling and regulation of NK cell responses at the maternal-fetal interface during pregnancy (Care et al., 2013; Lash et al., 2016; Ono et al., 2020). Mϕs have high plasticity and are usually classified into two subtypes, the proinflammatory M1-type (classically activated Mϕs) and the anti-inflammatory M2-type (alternatively activated Mϕs), depending on the tissue microenvironment (Gordon and Martinez, 2010). It is widely recognized that dMϕs are predominantly the M1 type to create an inflammatory microenvironment at the beginning of pregnancy and initiation of delivery, whereas dMϕs are predominantly the M2 type to induce immune tolerance during the maintenance of pregnancy (Brown et al., 2014). It has been reported that impaired polarization of dMϕs in early pregnancy can lead to RSA (Li et al., 2018; Shimada et al., 2018; Tsao et al., 2018).

SEC5 (also known as EXOC2) is a component of the exocyst complex and is involved in tank-binding kinase 1 (TBK1)-dependent type I interferon innate immune responses against viral infections (Chien et al., 2006; Ishikawa et al., 2009). Our previous studies demonstrated that SEC5 regulates not only macrophage phagocytosis and antifungal innate immune responses by interacting with inositol 1,4,5-trisphosphate receptor (InsP3R) (Yang et al., 2018) but also trophoblast cell migration and invasion through the integrin/Ca^2+^ signaling pathway (Gu et al., 2020). As the expression of SEC5 has been comprehensively detected in human villus and decidual tissues in early pregnancy (Gonzalez et al., 2014), we hypothesized that SEC5 might also participate in dMϕ polarization and that its dysfunction might lead to early pregnancy loss. Thus, in the present study, the SEC5 expression level in dMϕs of RSA patients was determined, and the role of SEC5 in M2 polarization of Mϕs was investigated using a human THP-1-derived Mϕ model *in vitro* and a mouse model of LPS-induced pregnancy loss *in vivo*.

## Materials and methods

### *In vitro* differentiation and treatment of human THP-1 cells

The human monocytic cell line THP-1 was purchased from ATCC. The cells were cultured in RPMI-1640 (Gibco, Thermo Fisher Scientific, United States) containing 10% heat-inactivated FBS (Gibco) and 0.055 mM 2-mercaptoethanol (Gibco) in a 5% CO_2_ atmosphere at 37°C. THP-1 cells were induced to differentiate into Mϕs as previously described (Chanput et al., 2014b; Huang et al., 2019b) with modifications. Briefly, the cells were treated with 200 ng/ml phorbol 12-myristate 13-acetate (PMA, Sigma, United States) followed by 24 h of incubation in RPMI-1640 medium to obtain a macrophage-like M0 state. Then, the M0-Mϕs were treated with fresh medium supplemented with 20 ng/ml IL-4 and 20 ng/ml IL-13 (PeproTech, Inc. United States) to induce differentiation into the M2 phenotype. siRNA specifically against SEC5 (SEC5 siRNA: 5′-GGUCGGAAAGACAAGGCAGdTdT-3′) and negative control siRNA (NC: 5′-UUC​UCC​GAA​CGU​GUC​ACG​UTT-3′) were synthesized by Shanghai GenePharma Co., Ltd. Transfections were performed using Lipofectamine 2000 according to the manufacturer’s instructions (Invitrogen, Thermo Fisher Scientific). For generation of polyclonal cell lines stably overexpressing SEC5 or a negative control, a lentivirus constructed and packaged by Shanghai Genechem Co., Ltd. was used to infect THP-1 cells. Cells expressing the Ubi-MCS-3FLAG-CBh-gcGFP vector (V) or expressing the recombinant SEC5 gene (SEC5) were grown in medium supplemented with puromycin at 1 μg/ml for approximately 14 days to eliminate uninfected cells. Quantitative real-time PCR and western blotting analyses were employed to determine the SEC5 expression level. Then, the selected cells continued to be grown in medium supplemented with puromycin at 0.5 μg/ml.

### Collection of human decidual tissue samples

Human decidual tissues from 12 RSA patients (RSA, 6–10 weeks of gestation) and 15 healthy pregnant women in the first trimester (control, 6–9 weeks of gestation) were collected at the Department of Gynecology and Obstetrics, the Second Hospital of Tianjin Medical University (Tianjin, China). These collected decidual tissues were immediately washed several times with sterile, glucose-free PBS solution until there were no obvious blood clots. Then, the tissues were immersed in ice-cold RPMI-1640 medium. Cells were harvested from the tissues or the tissues were fixed within 3 h. Current pregnancy losses of the RSA patients were objectively confirmed by transvaginal ultrasound examination. Patients with classical risk factors, including abnormal parental karyotypes, uterine anatomical abnormalities, infectious diseases, luteal phase defects, diabetes mellitus, thyroid dysfunction, and hyperprolactinemia, were excluded from this study. In parallel, control women who had no history of miscarriage and were undergoing legal, voluntary terminations of early pregnancy were enrolled and evaluated for classical risk factors for early pregnancy loss. The sample collection for this study was approved by the Medical Ethics Committees of The Second Hospital of Tianjin Medical University (KY 2017K002) and Shanghai Institute for Biomedical and Pharmaceutical Technologies (Ref # PJ 2018-17). All samples were collected after informed consent was obtained. No significant differences in the average age or gestational week at sampling were observed between the RSA patients and the control women (Supplementary Table S1).

### Isolation of human decidual macrophages

DMϕs were isolated as previously described (Houser et al., 2011; Jiang et al., 2018; Zhen et al., 2021). Briefly, tissues were washed and crushed into small pieces with a tissue crusher (Gentle MACS Dissociator, Miltenyi Biotec, Germany). Pieces of decidual tissues were digested in 3 mg/ml collagenase Type IV (Gibco, United States), 100 IU/ml DNase I (Sigma–Aldrich, United States), 100 IU/ml penicillin and 100 IU/ml streptomycin (Gibco) at 37°C for 30 min. Subsequently, the decidual cells that were released were filtered through 100-, 200-, and 400-mesh sieves (Corning, United States). To exclude any remaining red blood cells, the filtered cells were incubated with red blood cell lysis buffer (BD Biosciences, United States). DMϕs were obtained with anti-CD14 antibodies conjugated to magnetic beads (Miltenyi Biotec). The purity of the (CD45^+^ CD14^+^) dMϕs, which was detected by flow cytometry, was approximately 90%.

### Isolation and differentiation of bone marrow-derived macrophages

Primary BMDMs of C57BL/6 mice were prepared as previously described (Jia et al., 2014). In brief, bone marrow cells were aseptically collected from female mice aged 6–8 weeks (purchased from SIPPR/BK Laboratory Animal Company, Shanghai, China). The mice were sacrificed, and the collection area was sterilized with 75% ethanol. Then, the cells were isolated by flushing the mouse leg bones with phosphate-buffered saline (PBS). The cells were incubated in red blood cell lysis buffer (Solarbio, Beijing, China) before centrifugation. The cells were then cultured for 7 days in DMEM containing 20% FBS, 0.055 mM 2-mercaptoethanol (Gibco), antibiotic-antimycotic (Gibco), and 30% conditioned medium from L929 cells expressing macrophage colony-stimulating factor (M-CSF). Nonadherent cells were removed.

### Quantitative real-time PCR

Total RNA was extracted with TRIzol Reagent (Invitrogen, MA, United States) and then reverse transcribed into cDNA (TaKaRa Bio, Inc. Japan) according to the manufacturer’s instructions. The synthesized cDNA was amplified with specific primers (Supplementary Table S2) and SYBR Green (TaKaRa Bio, Inc.) using a LightCycler 480 II real-time fluorescence quantitative PCR system (Roche, Basel, Switzerland). Triplicate samples were examined for each condition. The relative mRNA expression level was calculated using the 2^−ΔΔCt^ method.

### Flow cytometry assay

For antibody staining, THP-1 cells were treated as indicated, washed, and incubated with CD206-PE and CD11b-FITC antibodies (BioLegend, United States) on ice for 30 min. Flow cytometric analysis was performed on a BD LSRFortessa system (BD Biosciences, United States), and the data were analyzed with FlowJo version 7.6.1 software. Inflammatory cytokines in the lysate of mouse uterine tissues were measured with the LEGENDplex Mouse Inflammation Panel (BioLegend, United States) according to the manufacturer’s instructions, and the data were analyzed with Qognit software.

### Immunofluorescence staining

THP-1 cells were differentiated as mentioned above and placed on coverslips (Fisher Scientific). The cells were fixed using 4% PFA in DPBS, followed by blocking and permeabilization with 0.1% Igepal (Sigma–Aldrich, Inc.) in DPBS with 2% BSA (Amresco, OH, United States). Primary antibodies diluted in DPBS with 2% BSA (Supplementary Table S3) were applied overnight at 4°C. The cells were subsequently washed four times with DPBS before being incubated with the appropriate secondary antibodies (Invitrogen, Thermo Fisher Scientific) diluted in 2% BSA in DPBS. The coverslips were washed four times with DPBS before being mounted on slides using mounting solution (Thermo Fisher Scientific). Confocal images were acquired with a Nikon A1R confocal system. For the decidual tissue slides, immunofluorescence staining of formalin-fixed and paraffin-embedded 5 μm sections was performed. After deparaffinization and rehydration, heat-induced antigen retrieval was performed by microwaving in 0.1 M sodium citrate (pH 6.0), and then, the slides were allowed to cool to room temperature. Endogenous peroxidase activity was blocked with 3% hydrogen peroxide for 10 min. The slides were blocked with 10% normal donkey serum in PBS-T (PBS +0.1% Triton X-100) for 1 h at room temperature and incubated with primary antibodies diluted in 5% BSA/PBS-T overnight at 4°C. After being washed twice with PBS-T for 10 min and then PBS for 10 min, the slides were incubated with Cy3-conjugated anti-rabbit (2.5 μg/ml) or Alexa Fluor 488-conjugated anti-mouse (2 μg/ml) secondary antibody at 37°C for 30 min. Next, the slides were washed, stained with Hoechst 33,342 (Invitrogen, Thermo Fisher Scientific), and mounted.

### Coimmunoprecipitation

THP-1 cells in the M0 or M2 state of differentiation as mentioned above were lysed using 1 × DPBS containing 1% Triton X-100. Rabbit SEC5 or STAT6 antibody (10 µg) or an equivalent amount of rabbit immunoglobulin G (IgG) isotype control (Sigma; #18140) was conjugated with 30 μL of a 50% slurry of protein G agarose resin according to the manufacturer’s instructions (Yeasen; #36405ES08). Then, the antibody-conjugated agarose resin was incubated with protein lysates (1 mg of protein) with gentle agitation for 2 h. The beads were retained after three washes with PBS. Next, 1× loading buffer was added to dissociate immunoprecipitates, and western blotting was performed accordingly.

### Protein extraction and western blot analysis

For preparation of whole-cell extracts, cells were washed with ice-cold PBS, incubated with RIPA lysis buffer (Sangon Biotech, Shanghai, China) containing 1 mM PMSF and protease inhibitor cocktail (Selleck, Shanghai, China) on ice, and then homogenized with an ultrasonic cell disruptor. The supernatant of the cell and tissue lysates was centrifuged at 12,000 × g for 15 min at 4°C. Cell nuclear protein was extracted using a Nucleoprotein Extraction Kit (Sangon Biotech) according to the manufacturer’s instructions. Approximately 50 μg of protein from each sample was subjected to SDS–PAGE, and the separated proteins were transferred to nitrocellulose membranes (Merck Millipore, Darmstadt, Germany). Blots were incubated with the appropriate primary antibodies diluted in TBST (containing 0.1% Tween 20 and 2% BSA) for 1 h at room temperature. Then, the blots were washed and incubated with appropriate secondary antibodies and detected using an Odyssey CLx Imaging System (LI-COR, Nebraska, United States).

### *In vitro* coculture model and transwell assay

After coculturing with THP1-derived Mϕs for 48 h, the alteration in morphology of HTR-8/SVneo cells was observed, and cells with more than two pseudopodia were counted as morphological changes. Cells in a total of 3 independent fields of view were counted in each group, for a total of at least 300 cells. HTR-8/SVneo cell invasion assay was performed using a BD BioCoat™ Matrigel™ Invasion Chamber (BD Biosciences, New Jersey, United States) according to the manufacturer’s instructions. A total of 1 ×10^5^ cells were seeded into the upper compartment of the prepared inserts, and medium with 25% FBS was added to the lower compartment to induce migration. After 24 h of incubation at 37°C with 5% CO_2_, the cells remaining inside the inserts were removed using a cotton swab. The membranes were then fixed with 4% paraformaldehyde, stained with 0.1% crystal violet (Sangon Biotech, Co., Ltd. Shanghai, China), and washed with ddH_2_O. After air-drying the samples, ten independent fields for each group were captured with a Nikon inverted microscope. Furthermore, the whole membranes containing all the stained cells were dissolved in methanol at 4°C for 10 min and mixed, and the absorbance at a wavelength of 560 nm was measured using a UV spectrophotometer. The number of invaded cells was determined by comparing the OD560 values. The experiments were repeated three times for each group under the same conditions.

### Mouse model of LPS-induced pregnancy loss

Adult male and female C57BL/6 mice (6–8 weeks old) were purchased from SIPPR/BK Laboratory Animal Company (Shanghai, China). Heterozygous SEC5-deficient (SEC5^−/+^) mice, constructed using CRISPR/Cas9, were purchased from Cyagen Biosciences (Suzhou, China). In short, SEC5 knockout was generated by pronuclear injection of C57BL/6 zygotes with 20 ng/μL Cas9 mRNA and two sgRNAs (10 ng/μL each; sgRNA 1 (5′-AAG​GTT​GTA​TAC​CTA​GAG​AC -3′) and sgRNA2 (5′-AAT​TCT​AGA​ACT​TTG​CCC​GC-3′)). SEC5^−/+^ mice were bred and genotyped. Genotypes were confirmed by PCR using the following primers: 5′-AAG​TCA​GGG​GAG​TAA​AGT​ACA​CAC-3′ and 5′-CTC​GTT​ATC​TTT​CAC​TGC AGTATCT-3’. All experiments were carried out in accordance with standard laboratory animal care protocols that were approved by the Institutional Animal Care Committee of Shanghai Institute for Biomedical and Pharmaceutical Technologies (#2018-14). Female mice were mated in natural cycling with males. Mice were inspected every morning for vaginal plugs. The day of vaginal plug detection was designated GD 0.5. Pregnant females were intraperitoneally injected with 250 μg/kg LPS (Sigma–Aldrich, St. Louis, MO, United States) at GD 7.5 to induce abortion. The control group was administered 100 μL of sterile saline solution. All mice were sacrificed at 24 h or 48 h after LPS treatment.

### Statistical analysis

At least three biological replicates were performed for all experiments unless otherwise indicated. Student’s t test was used for statistical analyses of paired observations. Differences between means were accepted as statistically significant at the 95% level (*p* < 0.05).

## Results

### SEC5 expression level is significantly decreased in the dMϕs of recurrent spontaneous abortion patients

**FIGURE 1:**
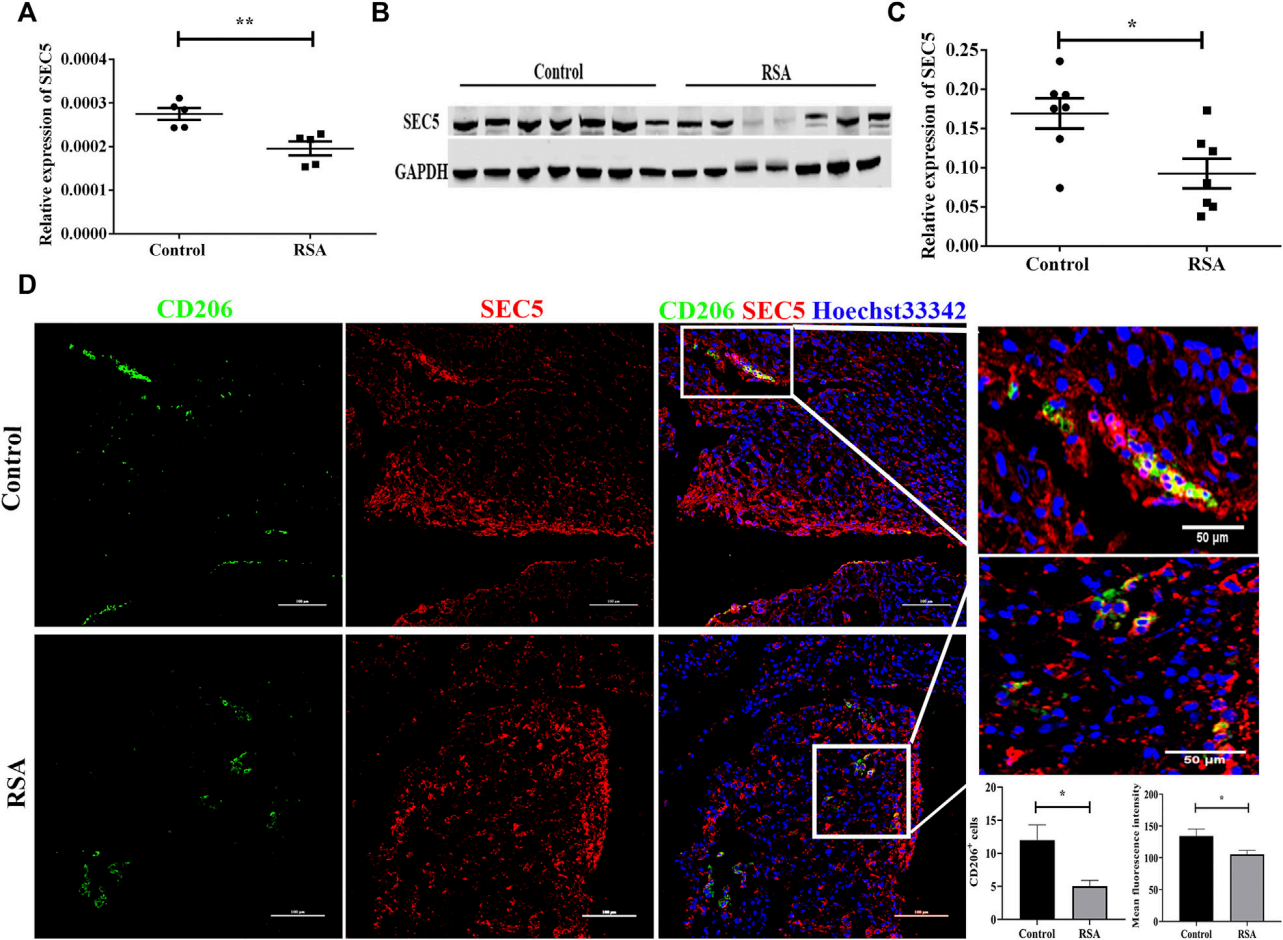
SEC5 expression is decreased in the dMϕs of patients with RSA. **(A)** SEC5 mRNA levels in dMϕs of healthy pregnant women (control, *n* = 5) and patients with RSA (RSA, *n* = 5) determined by qRT–PCR. **(B)** Representative images of the western blotting assay. **(C)** SEC5 protein expression levels in dMϕs of healthy pregnant women (control, *n* = 7) and patients with RSA (RSA, *n* = 7) detected by western blotting (normalized to the expression of GAPDH). **(D)** Distribution of SEC5 protein expression in human decidual tissues of healthy pregnant women (control, *n* = 3) and patients with RSA (RSA, *n* = 3) in the first trimester determined by immunofluorescence staining analysis. The histograms represent the statistical results of CD206^+^ cells per microscopic field, and the mean fluorescence intensity values for SEC5 expression in CD206^+^ cells, respectively. CD206: staining of CD206 (green, marker of M2-subtype macrophages); SEC5: staining of SEC5 (red); Hoechst 33,342: nuclear staining (blue). The results of **(A)** and **(C)** are expressed as the mean ± SEM of at least three independent experiments. *p < 0.05, **p < 0.01.

CD14^+^ dMϕs were isolated from decidual tissues of RSA patients and healthy pregnant women using immunomagnetic beads. The SEC5 expression level in dMϕs was subsequently determined by qPCR and western blot analyses. The results showed that the expression of SEC5 was significantly decreased in dMϕs of RSA patients (RSA) compared to the expression level in healthy pregnant women (control) (Figures 1A−C). Immunofluorescence costaining showed that SEC5 was widely expressed in human decidual tissues in early pregnancy and abundantly localized in M2-subtype (CD206^+^) macrophages (Figure 1D) and (Pan-CK^+^) trophoblasts (Supplementary Figure S1). As expected, CD206^+^ macrophage cells were significantly reduced in the decidua of RSA patients. In addition, the average fluorescence intensity of SEC5 (red) expressed in CD206^+^ macrophage cells (green) was obviously weaker in the decidua of RSA patients than in that of control women (Figure 1D).

### SEC5 induced M2 polarization of human and mouse Mϕs *in vitro*

**FIGURE 2:**
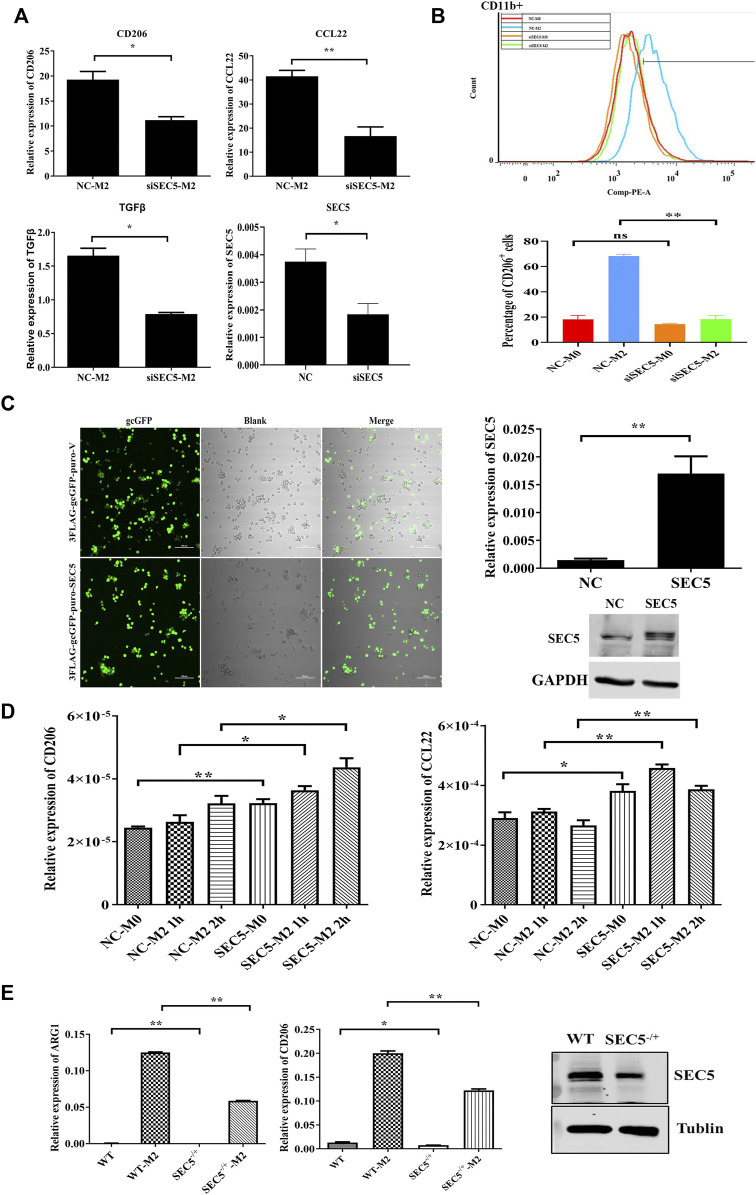
SEC5 promotes M2 polarization of macrophages induced by IL-4 and IL-13 *in vitro*. **(A)** Expression levels of M2 polarization markers (CD206, CCL22 and TGFβ) determined by qRT–PCR in THP-1 Mϕs stimulated with IL-4 and IL-13 for the indicated periods. NC-M2: M0-Mϕs transfected with the negative control and stimulated with IL-4 + IL-3; siSEC5-M2: M0-Mϕs transfected with SEC5-specific siRNA and stimulated with IL-4 + IL-3; NC: M0-Mϕs transfected with the negative control; siSEC5: M0-Mϕs transfected with SEC5-specific siRNA. **(B)** Flow cytometry demonstrated the cell surface expression of CD11b-FITC and CD206-PE on THP-1 Mϕs. We analyzed the percentage of CD206-positive cells in the CD11b-positive cell population. **(C)** Left: Representative images of cultured THP-1 cells transfected with the pCDNA3.0-GFP-Flag vector plasmid (3FLAG-gcGFP-puro-V) or the pCDNA3.0-GFP-Flag-SEC5 plasmid (3FLAG-gcGFP-puro-SEC5); Right: Expression level of SEC5 in THP1 cells detected by qRT–PCR and western blotting analysis. NC: M0-Mϕs transfected with the pCDNA3.0-GFP-Flag vector plasmid; SEC5: M0-Mϕs transfected with the pCDNA3.0-GFP-Flag-SEC5 plasmid. **(D)** Expression levels of CD206 and CCL22 in SEC5-overexpressing THP-1 Mϕs determined by qRT–PCR. NC-M0: M0-Mϕs transfected with pCDNA3.0-GFP-Flag vector plasmid; NC-M2: M0-Mϕs transfected with the pCDNA3.0-GFP-Flag vector plasmid and stimulated with 10 ng/ml IL-4 + 10 ng/ml IL-13 for 2 h; SEC5-M0: M0-Mϕs transfected with the pCDNA3.0-GFP-Flag-SEC5 plasmid; SEC5-M2: M0-Mϕs transfected with the pCDNA3.0-GFP-Flag-SEC5 plasmid and stimulated with 10 ng/ml IL-4 + 10 ng/ml IL-13 for 2 h; **(E)** Effects of SEC5 deficiency on M2 polarization of mouse BMDMs. Expression levels of the M2 markers ARG1 and CD206 in mouse BMDMs determined by qRT–PCR. SEC5 expression in BMDMs was determined by western blotting analysis. WT: BMDMs isolated from wild-type mice. SEC5^−/+^: BMDMs isolated from heterozygous SEC5-deficient mice. WTM2 and SEC5^−/+^ M2 cells were treated with IL-4 and IL-13 for 24 h. Data are shown as the mean ± SEM of three independent experiments. *p < 0.05, **p < 0.01.

The immortalized human peripheral blood monocyte cell line THP-1 was used to establish a human M0-type Mϕ model (M0) *via* treatment with PMA, and the M0-type Mϕs were treated with IL-4 and IL-13 to generate M2-type Mϕs (M2) *in vitro* (Chanput et al., 2014a; Huang et al., 2019a). SEC5 expression in THP1-derived Mϕs was down- or upregulated by transfection with a specific siRNA (siSEC5) or recombinant pCDNA3.0-GFP-Flag-SEC5 plasmid (SEC5-M0/SEC5-M2), respectively. The expression levels of the M2 phenotype markers CD206, CCL22, and TGFβ were detected to evaluate the effect of SEC5 expression on M2 polarization of human Mϕs *in vitro*. The results showed that the SEC5 expression level in THP1-derived Mϕs was effectively knocked down by siSEC5, and the reduction in SEC5 expression was accompanied by significantly decreased CD206, CCL22, and TGFβ expression levels (Figure 2A), indicating that the downregulation of SEC5 expression had an inhibitory effect on M2 polarization. To verify this finding, we measured the M2 macrophage cell surface marker CD206 *via* flow cytometry and found that SEC5 knockdown significantly reduced the cell surface expression of CD206 on CD11b^+^ THP-1 cells (Figure 2B). Moreover, the SEC5 expression level in THP-1-derived Mϕs transfected with the pCDNA3.0-GFP-Flag-SEC5 plasmid was obviously increased (Figure 2C), and upregulation of SEC5 expression led to increased CD206 and CCL22 expression levels (Figure 2D), suggesting a stimulatory effect of SEC5 on M2 polarization. Consistently, it was also observed that the M2 polarization of primary BMDMs derived from SEC5^−/+^ mice was significantly reduced compared to that of BMDMs from WT mice (Figure 2E), suggesting an inhibitory effect of decreased SEC5 expression on the M2 polarization of mouse Mϕs *in vitro*. Furthermore, SEC5 expression in mouse BMDMs was significantly reduced by transfection with siSEC5, and downregulation of SEC5 expression led to significantly decreased ARG1 and CD206 expression, two phenotypic markers of M2-type Mϕs in mice (Supplementary Figure S2).

### Phosphorylated STAT6 levels are reduced in both SEC5 knockdown human Mϕs and dMϕs of recurrent spontaneous abortion patients

**FIGURE 3:**
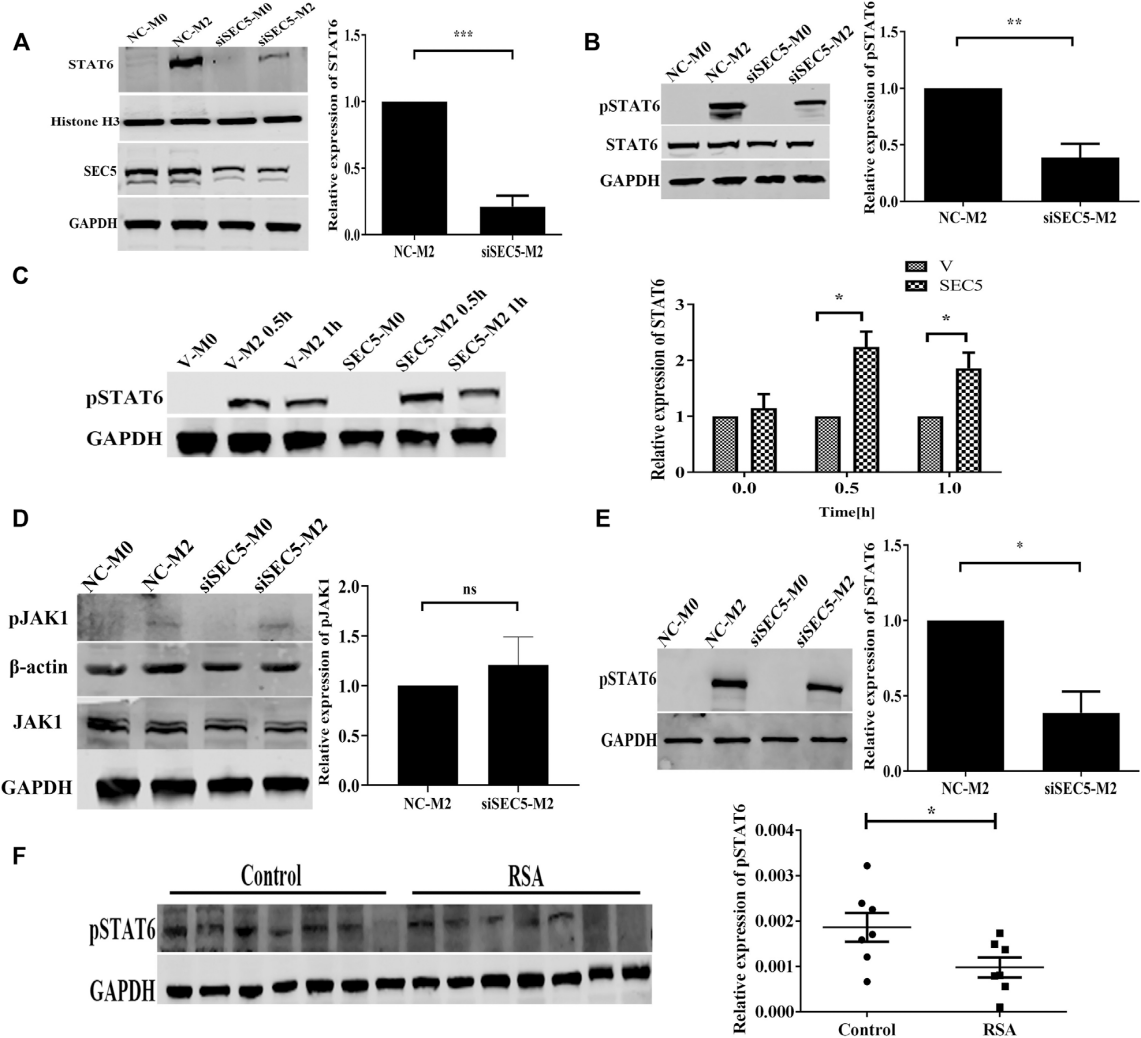
Phosphorylated STAT6 levels in SEC5 knockdown Mϕs and dMϕs of patients with RSA. **(A)** Upper: expression level of STAT6 in the nuclei of THP1-derived Mϕs examined by western blotting; lower: validation of downregulated SEC5 expression in THP1-derived Mϕs transfected with SEC5-siRNA. NC-M0: M0-Mϕs transfected with the negative control; NC-M2: M0-Mϕs transfected with the negative control and stimulated with IL-4 and IL-13; siSEC5-M0: M0-Mϕs transfected with SEC5-specific siRNA; siSEC5-M2: M0-Mϕs transfected with SEC5-specific siRNA and stimulated with IL-4 and IL-13. **(B)** Cytoplasmic levels of total STAT6 and phosphorylated STAT6 (pSTAT6) in THP1-derived Mϕs determined by western blotting. NC-M0/NC-M2/siSEC5-M0/siSEC5-M2: same as in **(A) (C)** Expression level of phosphorylated STAT6 (pSTAT6) in SEC5-overexpressing THP1-derived Mϕs detected by western blotting. v-M0: M0-Mϕs transfected with vector plasmid; v-M2 0.5 h/1 h: M0-Mϕs transfected with vector plasmid and stimulated with IL-4 + IL-13 for 0.5 or 1 h; SEC5-M0: M0-Mϕs transfected with pCDNA3.0-GFP-Flag-SEC5 plasmid; SEC5-M2 0.5 h/1 h: M0-Mϕs transfected with pCDNA3.0-GFP-Flag-SEC5 plasmid and stimulated with IL-4 + IL-13 for 0.5 or 1 h. **(D)** Expression level of phosphorylated JAK1 (pJAK1) in THP1-derived Mϕs determined by western blotting. NC-M0/NC-M2/siSEC5-M0/siSEC5-M2: same as in **(A) (E)** Expression level of phosphorylated STAT6 (pSTAT6) in mouse BMDMs determined by western blotting. NC-M0: BMDMs transfected with the negative control; NC-M2: BMDMs transfected with the negative control and stimulated with IL-4 + IL-13; siSEC5-M0: BMDMs transfected with SEC5-specific siRNA; siSEC5-M2: BMDMs transfected with SEC5-specific siRNA and stimulated with IL-4- + -IL-13. **(F)** Expression level of phosphorylated STAT6 (pSTAT6) in dMϕs of patients with RSA detected by western blotting. Control: dMϕs of healthy pregnant women; RSA: dMϕs of patients with RSA. All experiments were independently repeated at least 3 times. *p < 0.05, **p < 0.01, ***p < 0.001.

Given that IL-4/IL-13-induced M2 polarization is mediated by STAT6 signaling (Gordon and Martinez, 2010), we investigated the effect of SEC5 on the activation of STAT6 in the M2 polarization of THP-1-derived human Mϕs. The amount of STAT6 translocated to the nucleus was obviously increased after M2 polarization of the THP-1-derived Mϕs but significantly reduced in the SEC5 knockdown Mϕs (Figure 3A). Although the cytoplasmic STAT6 protein expression level in THP-1-derived Mϕs was not affected by M2 polarization or SEC5 knockdown, the level of STAT6 phosphorylated at tyrosine 641 (Y641) (pSTAT6) among the total cytoplasmic proteins was significantly increased after M2 polarization but dramatically reduced in SEC5 knockdown M2 cells (Figure 3B). In contrast, the pSTAT6 level in SEC5-overexpressing THP-1-derived Mϕs was significantly increased after treatment with IL-4 and IL-13 (Figure 3C). However, neither total JAK1 protein nor phosphorylated JAK1 (pJAK1, Tyr1034/1035) was affected by downregulation of SEC5 expression (Figure 3D). In mouse BMDMs, the increased pSTAT6 level caused by M2 polarization was also markedly reduced by SEC5 knockdown (Figure 3E), indicating an inhibitory effect of decreased SEC5 expression on the phosphorylation of STAT6 protein in human and mouse Mϕs. Interestingly, it was found that the pSTAT6 level in dMϕs of RSA patients was also significantly decreased (Figure 3F).

### SEC5 interacts with STAT6 and is predominantly colocalized in the nucleus of M2-Mϕs

**FIGURE 4:**
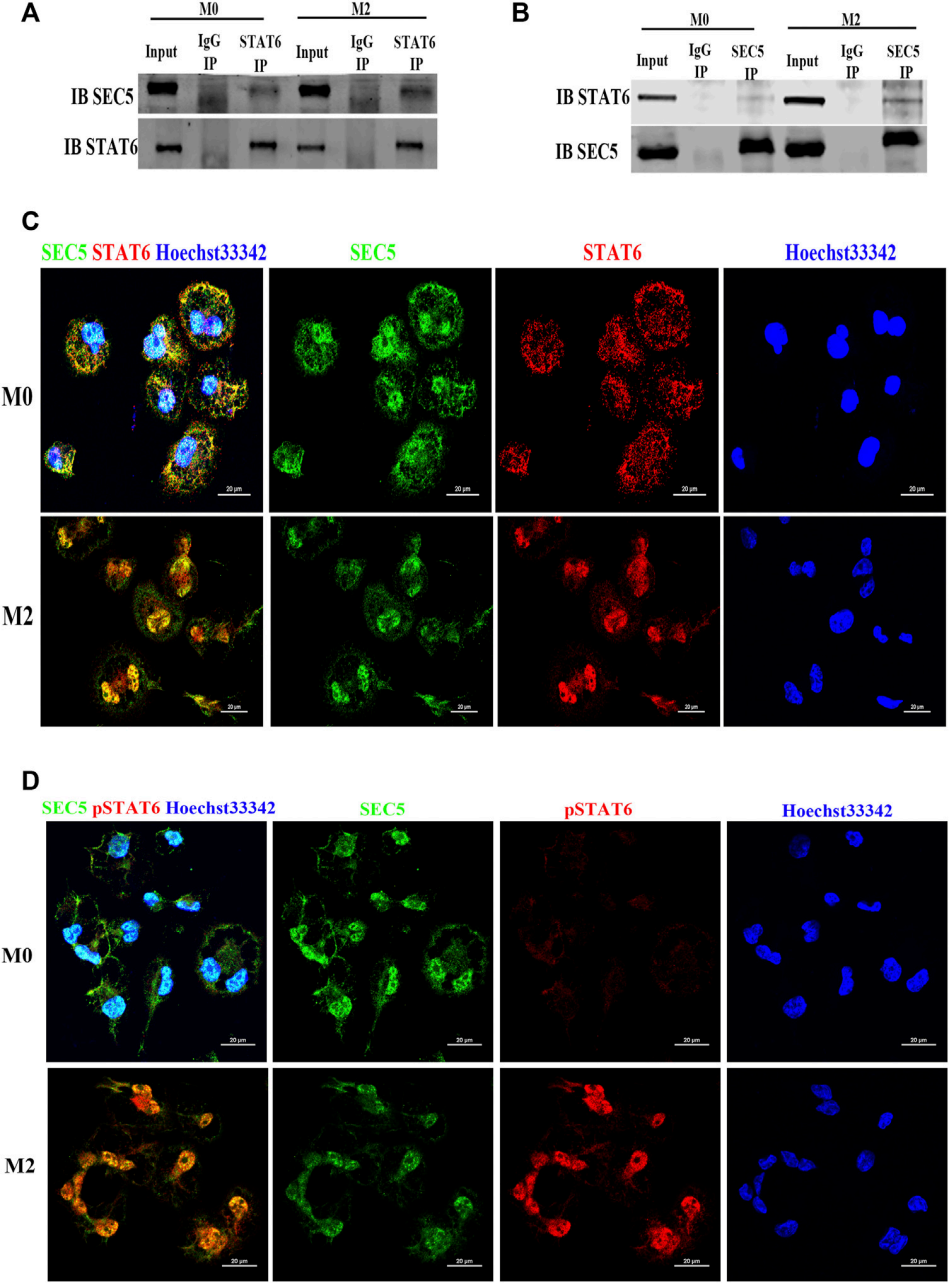
SEC5 interacts and colocalizes with STAT6 protein in human Mϕs. **(A)** The interaction between SEC5 and STAT6 in THP1-derived Mϕs was detected by immunoprecipitation (IP) assays in which samples were immunoprecipitated using anti-STAT6 antibody and immunoblotted with anti-SEC5 antibody. **(B)** The interaction between SEC5 and STAT6 in THP1-derived Mϕs was detected by IP assays using an anti-SEC5 antibody for immunoprecipitation and an anti-STAT6 antibody for immunoblotting. **(C)** Double immunofluorescence staining of SEC5 and STAT6 in THP1-derived Mϕs. **(D)** Double immunofluorescence staining of SEC5 and phosphorylated STAT6 (pSTAT6) in THP1-derived Mϕs. M0: M0 Mϕs; M2: M0 Mϕs treated with IL-4+IL-13.

As SEC5 regulated the activation of STAT6, we wondered whether SEC5 directly interacts with STAT6 in Mϕs. The results of coimmunoprecipitation (co-IP) assays showed that the SEC5 antibody precipitated STAT6 protein (Figure 4A) and the STAT6 antibody precipitated SEC5 protein (Figure 4B) in the THP-1-derived Mϕs, and the binding capacity was enhanced after M2 polarization induced by IL-4 and IL-13 (Figures 4A,B), indicating an interaction between SEC5 and STAT6 in human Mϕs. To further confirm this interaction, we determined the spatial distribution of SEC5 and STAT6 in THP-1-derived Mϕs *via* immunofluorescence staining assays. SEC5 colocalized with STAT6 in both the nucleus and cytoplasm of M0-Mϕs but predominantly in the nucleus of M2-Mϕs, as STAT6 was activated and translocated to the nucleus (Figure 4C). Furthermore, after M2 polarization, phosphorylated STAT6 (pSTAT6) protein signals were obviously enhanced and predominantly localized in the nucleus of Mϕs, and SEC5 protein signals were also colocalized with pSTAT6 (Figure 4D).

### Knockdown of SEC5 reverses the effect of M2-Mϕs on extravillous trophoblasts

**FIGURE 5:**
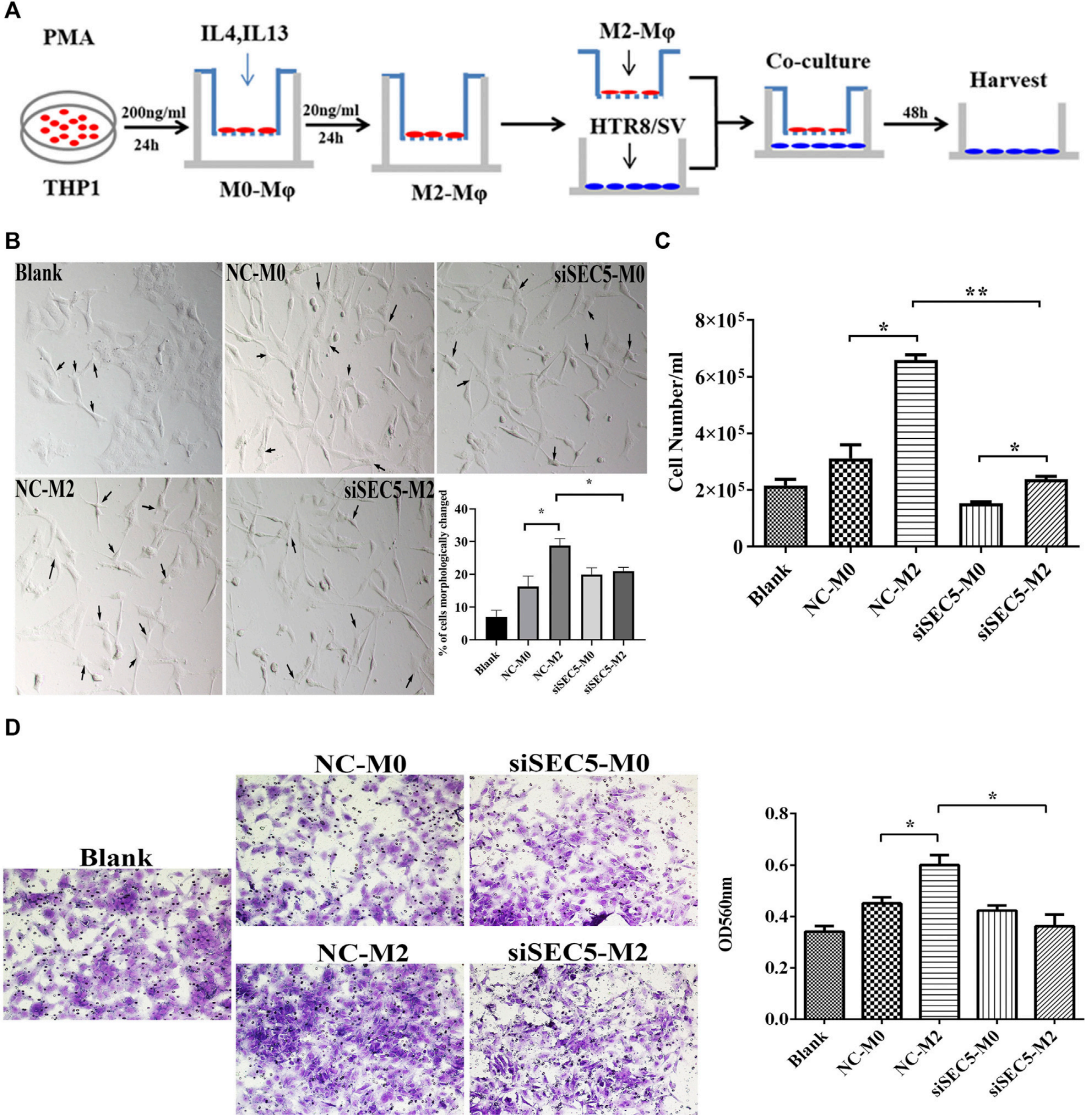
Downregulation of SEC5 expression reverses the stimulatory effects of M2-Mϕs on EVTs. **(A)** Schematic diagram of the coculture model of THP1-derived Mϕs and HTR-8/SVneo cells. **(B)** Representative bright field images of cultured HTR-8/SVneo cells and statistical results showing the percentage of cells with morphological changes (cells in a total of 3 independent fields of view were counted in each group, for a total of at least 300 cells). **(C)** Proliferation of HTR-8/SVneo cells detected with cell counting assays. **(D)** Invasion of HTR-8/SVneo cells detected with Transwell assays. Left: Representative images of a Matrigel invasion assay with crystal violet staining; Right: statistical analysis (based on the OD values at 560 nm) of the cells in each Transwell assay group that were stained after invasion and dissolved in methanol. Blank: HTR-8/SVneo cells were cultured in medium; NC-M0: HTR-8/SVneo cells were cocultured with M0-Mϕs transfected with NC; siSEC5-M0: HTR-8/SVneo cells were cocultured with M0-Mϕs transfected with SEC5-specific siRNA; NC-M2: HTR-8/SVneo cells were cocultured with M2-Mϕs transfected with NC; siSEC5-M2: HTR-8/SVneo cells were cocultured with M2-Mϕs transfected with SEC5-specific shRNA. All experiments were independently repeated at least 3 times. *p < 0.05, **p < 0.01.

It has been reported that M2-Mϕs promote the epithelial-to-mesenchymal transition (EMT), migration and invasion of extravillous trophoblasts (EVTs) by secreting granulocyte-colony stimulating factor (G-CSF) (Ding et al., 2021b). Thus, we evaluated whether downregulation of SEC5 expression would interfere with the effect of M2-Mϕs on EVT activities by using an *in vitro* coculture model (Figure 5A). After coculture with THP-1-derived M2-Mϕs, the percentage of HTR-8/SVneo cells, an immortalized human first trimester EVT line, with multiple pseudopodia was significantly increased (Figure 5B, black arrows). However, HTR-8/SVneo cells cocultured with SEC5 knockdown Mϕs were contracted and had elongated tentacles, and the percentage of cells with multiple pseudopodia was significantly decreased (Figure 5B). Consistent with the reported data, cell counting and Transwell assay results showed that the proliferation and invasion of HTR-8/SVneo cells cocultured with M2-Mϕs were significantly enhanced compared to those of HTR-8/SVneo cells cocultured with M0-Mϕs, and interestingly, such stimulatory effects were effectively weakened by downregulation of SEC5 expression in Mϕs (Figures 5C,D).

### Impaired SEC5 expression promotes LPS-induced pregnancy loss in mice

**FIGURE 6:**
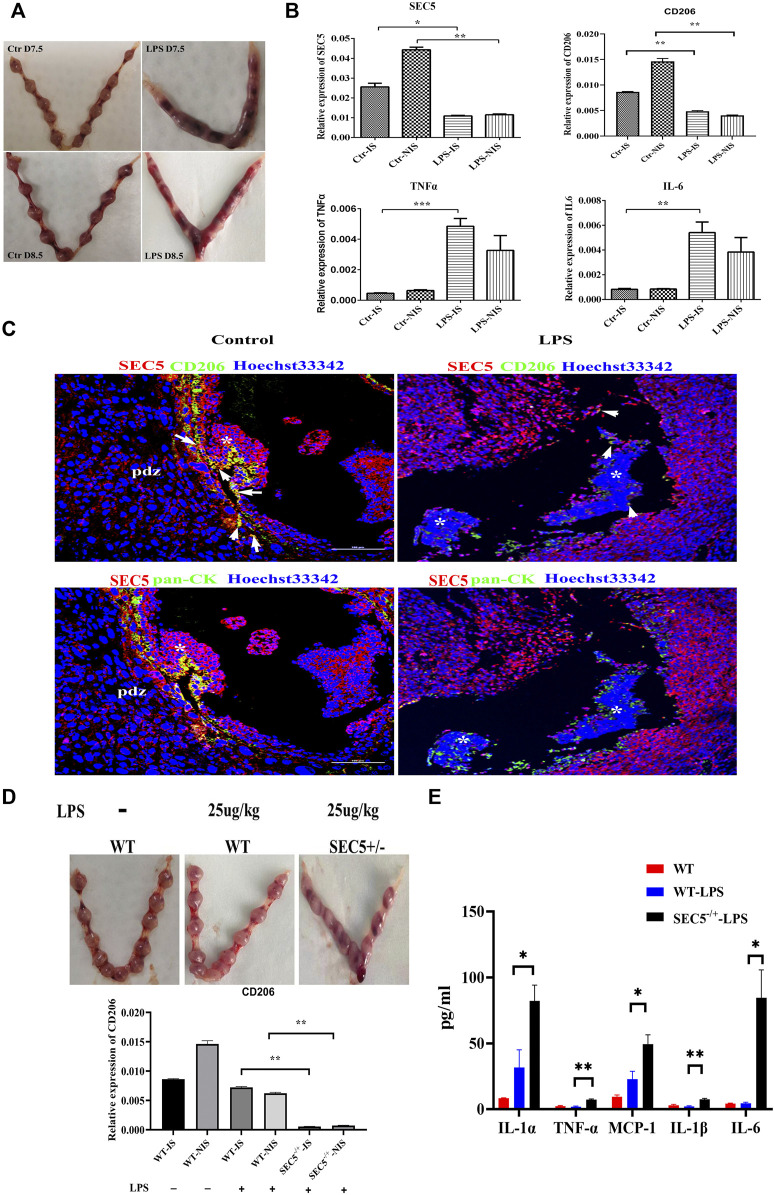
Uterine expression of SEC5 is decreased in mice with LPS-induced early pregnancy loss. **(A)** Representative images of the uterus (GD 7.5 and GD 8.5) from a saline-injected mouse (Ctr) and an aborted uterus (GD 7.5 and GD8.5) from an LPS-injected mouse (LPS) are shown. The embryo mortality rate induced by LPS (250 μg/kg) was 100%. **(B)** Uterine expression levels of SEC5, CD206, TNFα and IL-6 determined by qRT–PCR. Ctr-IS: implantation sites of the GD 7.5 saline-injected mice; Ctr-NIS: nonimplantation sites of the GD 7.5 saline-injected mice; LPS-IS: implantation sites of the GD 7.5 LPS-injected mice; LPS-NIS: nonimplantation sites of the GD 7.5 LPS-injected mice; **(C)** Representative images of immunofluorescence staining assays. Uterine tissues were collected from the GD 8.5 saline-injected mice (control) and the GD 7.5 LPS-injected mice (LPS). The white arrows indicate the areas where SEC5 and CD206 are colocalized. The asterisk indicates the remaining embryonic tissue. **(D)** Upper: representative images of uteri from wild-type (WT) and SEC5 knockout (SEC5^−/+^) pregnant mice treated with low-dose LPS (25 μg/kg); lower: uterine expression levels of CD206 determined by qRT–PCR. **(E)** The expression of proinflammatory factors in mouse uterine lysate was determined using LEGENDplex assays. *p < 0.05, **p < 0.01, ***p < 0.001.

It has been reported that administration of LPS to pregnant mice at an early gestational stage can cause pregnancy loss by destroying immune tolerance at the maternal-fetal interface (Aisemberg et al., 2007; Aisemberg et al., 2013; Zhou et al., 2017). According to previously described methods (Triggianese et al., 2016), we established an early pregnancy loss mouse model *via* a single intraperitoneal injection of LPS (250 μg/kg) on GD 6.5 (GD 0.5 = day of vaginal plug), and 100% fetal death was observed 24 h after LPS injection (GD7.5), followed by embryonic resorption 48 h after injection (GD8.5) (Figure 6A). qPCR results showed that the expression levels of CD206, IL-6 and TNFα were significantly increased in LPS-treated mice on GD 7.5, whereas the SEC5 expression level was distinctly decreased at both implantation sites (ISs) and nonimplantation sites (NISs) compared with the control GD7.5 mice (Figure 6B). Immunofluorescence costaining assays showed that the number of M2-Mϕs (CD206^+^, green) and the intensity of SEC5 signals (red) in uterine tissues in LPS-treated mice (LPS) seemed to be reduced on GD 8.5 compared to these parameters in normal mice (control) (Figure 6C). Moreover, EVTs (pancytokeratin^+^, green) invading uterine tissues were observed and were localized near Mϕs (Figure 6C). To investigate the role of SEC5 in LPS-induced early pregnancy loss, SEC5 gene deletion mice were constructed using CRISPR/Cas9 technology (Supplementary Figure S2). We intraperitoneally injected wild-type and SEC5^−/+^ mice with low doses of LPS (25 μg/kg) on GD 7.5. Uterine tissue from the pregnant SEC5^−/+^ mice showed embryonic death on GD 8.5, while the wild-type mice did not (Figure 6D). Furthermore, the expression levels of the proinflammatory cytokines IL-1α, TNFα, IL-1β, and IL-6 were significantly increased at the implantation sites in SEC5^−/+^ pregnant mice (Figure 6E).

## Discussion

**FIGURE 7:**
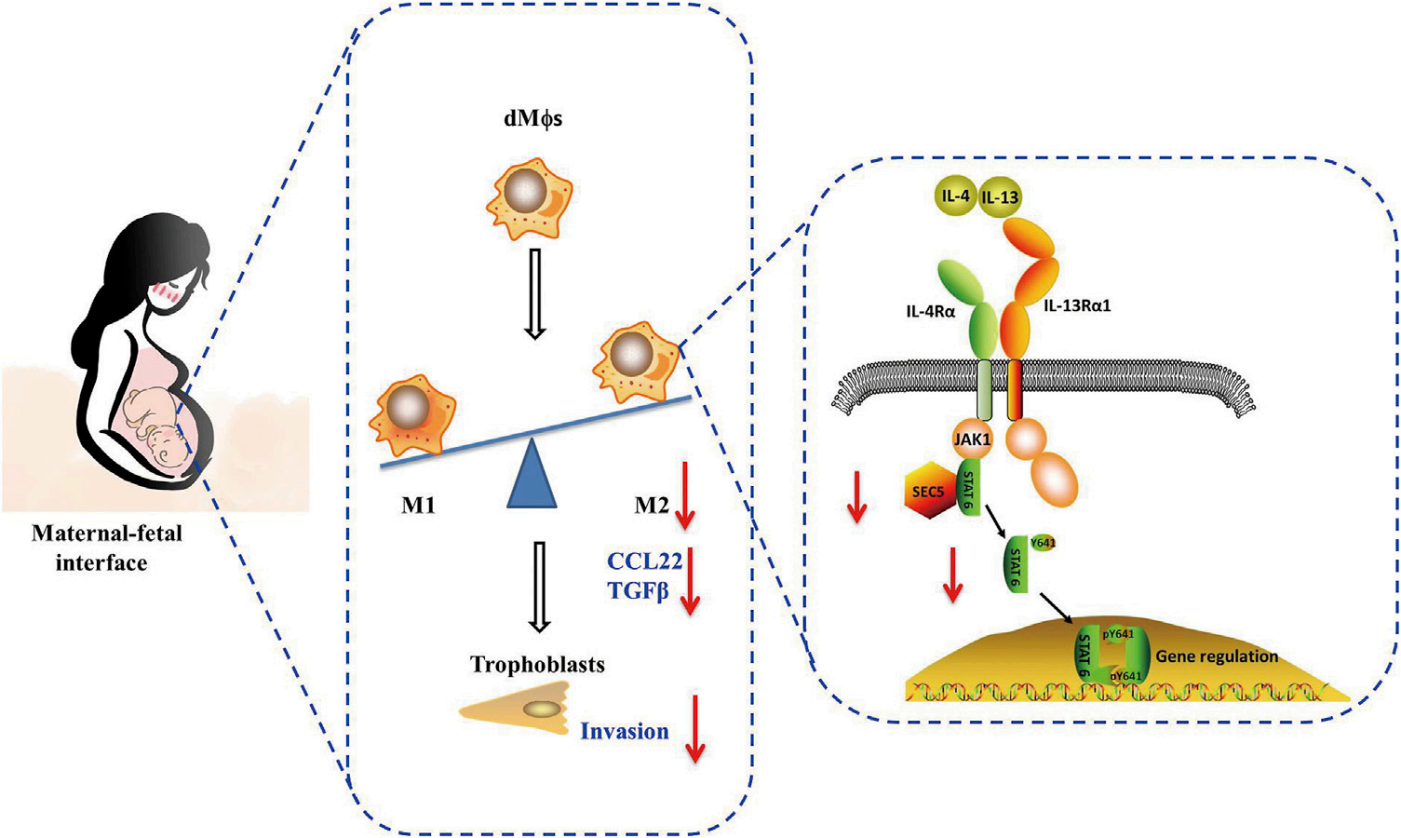
A schematic diagram of the impairment of macrophage M2 polarization induced by low SEC5 expression at the maternal–fetal interface in patients with RSA. SEC5 regulates STAT6 phosphorylation through its interaction with STAT6, thereby leading to macrophage M2 polarization.

This study demonstrated that the SEC5 expression level was significantly decreased both in dMϕs of RSA patients and in uterine tissues of pregnant mice with LPS-induced early pregnancy loss. Knockdown of SEC5 in Mϕs inhibited M2 polarization and STAT6 phosphorylation, whereas overexpression of SEC5 promoted M2 polarization and STAT6 phosphorylation in Mϕs. Increased pregnancy loss and uterine expression of inflammatory factors were observed in heterozygous SEC5-deficient (SEC5^−/+^) pregnant mice. Furthermore, SEC5 interacted with STAT6 protein in Mϕs, and the phosphorylated STAT6 (pSTAT6) level in dMϕs of RSA patients was also significantly reduced. The abundance of pSTAT6 protein was obviously increased in Mϕs, with a predominant distribution in the nucleus, after M2 polarization, and SEC5 protein was colocalized with pSTAT6. M2 polarization of Mϕs was accompanied by stronger stimulatory effects on the proliferation and invasion of human extravillous trophoblasts (EVTs) in a paracrine manner *in vitro*, and knockdown of SEC5 expression in Mϕs effectively reversed these effects (Figure 7).

Decidual macrophage polarization phenotypes undergo dynamic changes during different stages of gestation. Successful pregnancy depends on delicate synergism in M1/M2 polarization of dMϕs at the maternal-fetal interface to provide a balanced immune microenvironment for the fetus. During the peri-implantation period, dMϕ polarity is skewed toward the M1 subtype to induce the inflammatory response, whereas after the invasion of trophoblasts into the uterine stroma, dMϕ polarity tends toward the M2 subtype to support vasculature remodeling and a tolerant immune microenvironment until the initiation of parturition. Parturition is another inflammatory event, and M1 polarization of dMϕs benefits the expulsion of the baby and placenta (Brown et al., 2014). Aberrant changes in the polarization of dMϕs can affect the microenvironment at the maternal-fetal interface, leading to adverse pregnancy outcomes, such as RSA, preeclampsia and preterm labor (Zhang et al., 2017; Jena et al., 2019). Thus, deeper insight into the molecular mechanisms underlying abnormal dMϕ polarization would undoubtedly contribute to improvements in the clinical management of RSA.

Given that we previously found that SEC5 participates in the regulation of macrophage phagocytosis (Yang et al., 2018), as well as trophoblast invasion activity (Gu et al., 2020), we speculated that SEC5 might also be involved in the polarization of dMϕs during early pregnancy in humans. As expected, SEC5 protein signals were detected in various cells of human decidual tissues at early pregnancy, including M2-dMϕs and invasive trophoblasts, and the SEC5 expression level in the dMϕs of RSA patients was significantly reduced compared to that in healthy early pregnant women (Figure 1), preliminarily indicating that SEC5 deficiency might cause early pregnancy loss by inhibiting M2 polarization. Furthermore, we observed that, M2 polarization stimulated in THP-1 cells or BMDMs did not affect the expression level of SEC5 (Supplementary Figure S4), indicating that, down-regulated expression of SEC5 impaired the M2 polarization of decidual macrophages, which disrupted the immune homeostasis at the maternal-fetal interface.

As the T helper 2 (Th2) cell cytokines IL-4 and IL-13 can induce M2 polarization of human Mϕs (Gordon and Martinez, 2010; Moshkovits et al., 2015; Guo et al., 2019), in this study, we established M2 polarization of a human THP-1-derived Mϕ model *via* treatment with IL-4 and IL-13. Downregulation of SEC5 expression in THP-1-derived Mϕs inhibited M2 polarization, whereas increased SEC5 expression promoted M2 polarization (Figure 2). Interestingly, we found that down-regulation of SEC5 expression did not affect the expression of the M1 polarization marker CD80 in either stimulated or resting states. However, the expression levels of IL-6 and TNFα were significantly increased accompanied with the decrease of SEC5 expression (Supplementary Figure S5). Therefore, we speculated that SEC5 plays a specific role in M2, and that it may affect the signaling pathways that regulate the expression of IL-6 and TNFα, thereby directly affecting their expression.

T helper (Th) cells are another important immune cell population at the maternal-fetal interface, and the Type 1 Th cell (Th1)/Th2 shift plays an essential role in local immune regulation during pregnancy (Raghupathy, 2001; Sargent et al., 2006). As Th2 cells contribute to immune tolerance by secreting cytokines, including IL-4 and IL-13, it was reasonable for us to speculate that neighboring decidual Th2 cells might promote M2 polarization of dMϕs by secreting IL-4 and IL-13 at the maternal-fetal interface during early pregnancy.

The JAK/STAT signaling pathway is associated with the immune regulation of IL-4/IL-13 signaling (Darnell et al., 1994) and M2 polarization (Yang et al., 2021). JAK1 is activated following the binding of IL-4 to its receptor, which induces the assembly of an active receptor complex and the consequent phosphorylation of JAK1. Activated JAKs further transmit cytokine signals from membrane receptors, leading to the phosphorylation of STATs (Chen et al., 2011). Thus, we initially speculated that SEC5 might be involved in IL-4/IL-13-induced M2 polarization by regulating JAK activity. However, it was found that neither the total JAK1 protein nor phosphorylated JAK1 (Tyr1034/1035) levels were affected by the alteration of SEC5 expression levels in THP1-derived Mϕs, and no direct interaction between JAK and SEC5 was detected. Then, we focused on STAT6, a downstream molecule of JAK1.

STAT6 is required for innate and adaptive immune signaling in response to virus infection or signals from extracellular cytokines through JAK-dependent or JAK-independent pathways (Levy and Darnell, 2002; Chen et al., 2011). STAT6 plays important roles in multiple functions regulated by IL-4 and IL-13, including the differentiation of Th2 cells and production of IgE, chemokines, and mucus at sites of allergic inflammation (Gordon and Martinez, 2010). Moreover, IL-4 can regulate M2 polarization *via* a STAT6-dependent pathway in which IL-4 induces phosphorylation of the IL-4 receptor to recruit cytosolic STAT6. The recruited STAT6 is then phosphorylated on tyrosine 641 (Y641) by JAK1 upon M2 polarization, which results in nuclear translocation of STAT6 to activate its target genes (Murray, 2017; He et al., 2020). Here, we found that SEC5 bound to STAT6 in Mϕs and that the interacting proteins were predominantly colocalized in the nucleus after M2 polarization; furthermore, phosphorylated STAT6 levels were significantly decreased in both the SEC5 knockdown Mϕs and dMϕs of RSA patients (Figure 4), suggesting that SEC5 might be involved in the phosphorylation and translocation of STAT6 in Mϕs by direct interaction with STAT6.

Appropriate invasion of EVTs into decidual tissues is necessary for successful pregnancy, and dMϕs are located close to invading EVTs within the decidua (Brown et al., 2014). There is delicate crosstalk between EVTs and dMϕs during normal pregnancy. EVTs produce and secrete signaling molecules, such as HLA, to regulate the polarization of dMϕs (Wang et al., 2019; Zhang et al., 2020; Ding et al., 2021a). In addition, macrophages with different polarization states have different effects on the motility of EVTs (Jena et al., 2019; Ding et al., 2021b). It has been reported that M2-Mϕs promote the invasion of EVTs by secreting G-CSF (Ding et al., 2021b), whereas M1-dMϕs inhibit the proliferation and invasion of EVTs by secreting TNFα (Ning et al., 2016; Zhen et al., 2021). Interestingly, here, it was found that M2-Mϕs could enhance the proliferation and invasion of HTR-8/SVneo cells in a paracrine manner, consistent with the reported data, and downregulation of SEC5 expression in M2-Mϕs effectively reversed these stimulatory effects (Figure 5), suggesting that deficiency of SEC5 in dMϕs might cause early pregnancy loss, at least partially by affecting the normal proliferation and invasion of EVTs. Notably, SEC5 deficiency in EVTs also leads to reduced invasion of EVTs (Gu et al., 2020 ). Furthermore, we found that down-regulation of SEC5 in THP-1 reduced the mRNA expression of G-CSF (Supplementary Figure S6), indicating that SEC5 may regulate the invasion of EVTs by affecting the expression and secretion of G-CSF in M2-Mϕs.

LPS can induce M1 polarization of human Mϕs and has been used to establish an immune response-mediated mouse model of spontaneous abortion (Triggianese et al., 2016; Cui et al., 2021). In this study, a mouse model of LPS-induced abortion was applied to further explore the association between reduced SEC5 expression and early pregnancy loss in mice. Encouragingly, it was found that uterine expression levels of IL-6 and TNFα were significantly increased in mice with LPS-induced abortion and that the SEC5 expression level was distinctly decreased, accompanied by a reduced amount of uterine M2-Mϕs (Figure 6), indicating that LPS might interfere with the normal M1/M2 shift by downregulating SEC5 expression in uterine Mϕs at early pregnancy. Subsequently, the production and secretion of proinflammatory cytokines, such as IL-6 and TNFα, in uterine Mϕs are abnormally increased to attenuate EVT invasion and destroy the immune tolerance at the maternal-fetal interface, ultimately resulting in pregnancy failure. The LPS-induced mouse model of abortion is a suitable *in vivo* model for future investigation of the role of the SEC5/STAT6 molecular pathway in M2 polarization of uterine Mϕs.

In summary, these data indicate that SEC5 is involved in M2 polarization of macrophages by interacting with STAT6, and deficiency of SEC5 in decidual macrophages might lead to early pregnancy loss by interfering with EVT invasion and immunotolerance at the maternal-fetal interface, presenting a potential pathogenic mechanism of URSA caused by immune factors.

## Data Availability

The datasets presented in this study can be found in online repositories. The names of the repository/repositories and accession number(s) can be found in the article/Supplementary Material.

## Supplementary material

Supplementary Figure S1 Immunofluorescence staining of SEC5 and pan-CK in human decidual tissues of a healthy pregnant woman (control) and patients with RSA (RSA) in the first trimester. pan-CK: staining of pan-CK (green, marker of trophoblast cells); SEC5: staining of SEC5 (red); Hoechst 33342: nuclear staining (blue).

Supplementary Figure S2 Expression levels of Arginase 1 (ARG1) and CD206 in mouse BMDMs determined by qRT-PCR. NC-M2: BMDMs transfected with the negative control sequence of SEC5 and treated with IL-4 and IL-13; siSEC5-M2: BMDMs transfected with SEC5-specific siRNA and treated with IL-4 and IL-13; NC-M0: BMDMs transfected with the negative control; siSEC5-M0: BMDMs transfected with SEC5-specific siRNA.

Supplementary Figure S3 Genotyping of SEC5 heterozygous mice. Representative images of genotyping via PCR amplification. Heterozygotes: one band with 460 bp.

Supplementary Figure S4 Expression levels of SEC5 determined by qRT-PCR in THP-1 Ms and BMDMs stimulated with IL-4 and IL-13 for 24 h.

Supplementary Figure S5 Expression levels of M1 polarization markers (CD80, IL-6 and TNFα) determined by qRT-PCR in THP-1 Ms stimulated with LPS (20 ng/ml) and IFNγ (20 ng/ml) for 24 h. NC-M0: M0-Ms transfected with the negative control; siSEC5-M0: M0-Ms transfected with SEC5-specific siRNA; NC-M1: M0-Ms transfected with the negative control and stimulated with LPS and IFNγ; siSEC5-M1: M0-Ms transfected with SEC5-specific siRNA and stimulated with LPS and IFNγ.

Supplementary Figure S6 Expression levels of G-CSF and SEC5 determined by qRT-PCR in THP-1 Ms stimulated with IL-4 and IL-13. NC: M0-Ms transfected with the negative control; siSEC5: M0-Ms transfected with SEC5-specific siRNA; NC-M2: M0-Ms transfected with the negative control and stimulated with IL-4 + IL-3; siSEC5-M2: M0-M s transfected with SEC5-specific siRNA and stimulated with IL-4 + IL-3.
