# Supplementary figures and images for: SEC5 is involved in M2 polarization of macrophages via the STAT6 pathway, and its dysfunction in decidual macrophages is associated with recurrent spontaneous abortion
Source: Front Cell Dev Biol. 2022 Oct 14;10:891748. doi: 10.3389/fcell.2022.891748 (PMC9614079; doi:10.3389/fcell.2022.891748)

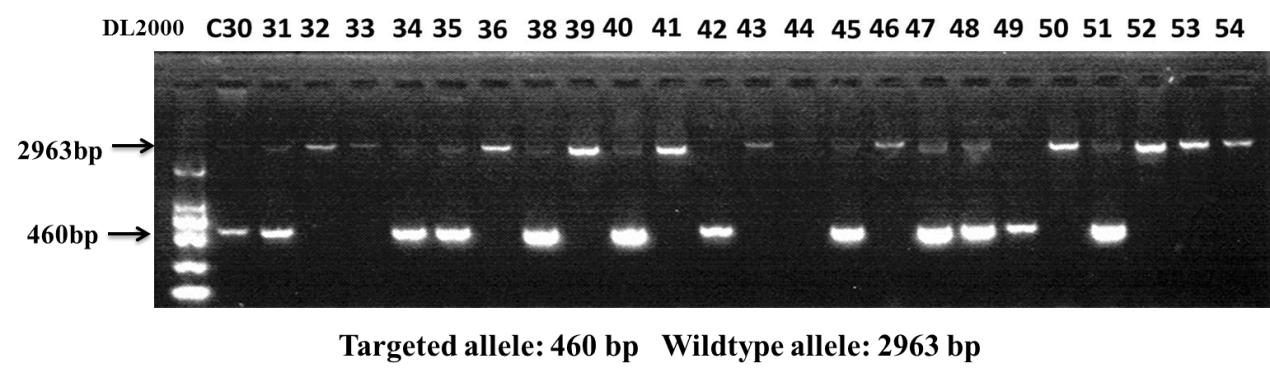

Supplement: Supplementary file 1 [file Image3.JPEG]

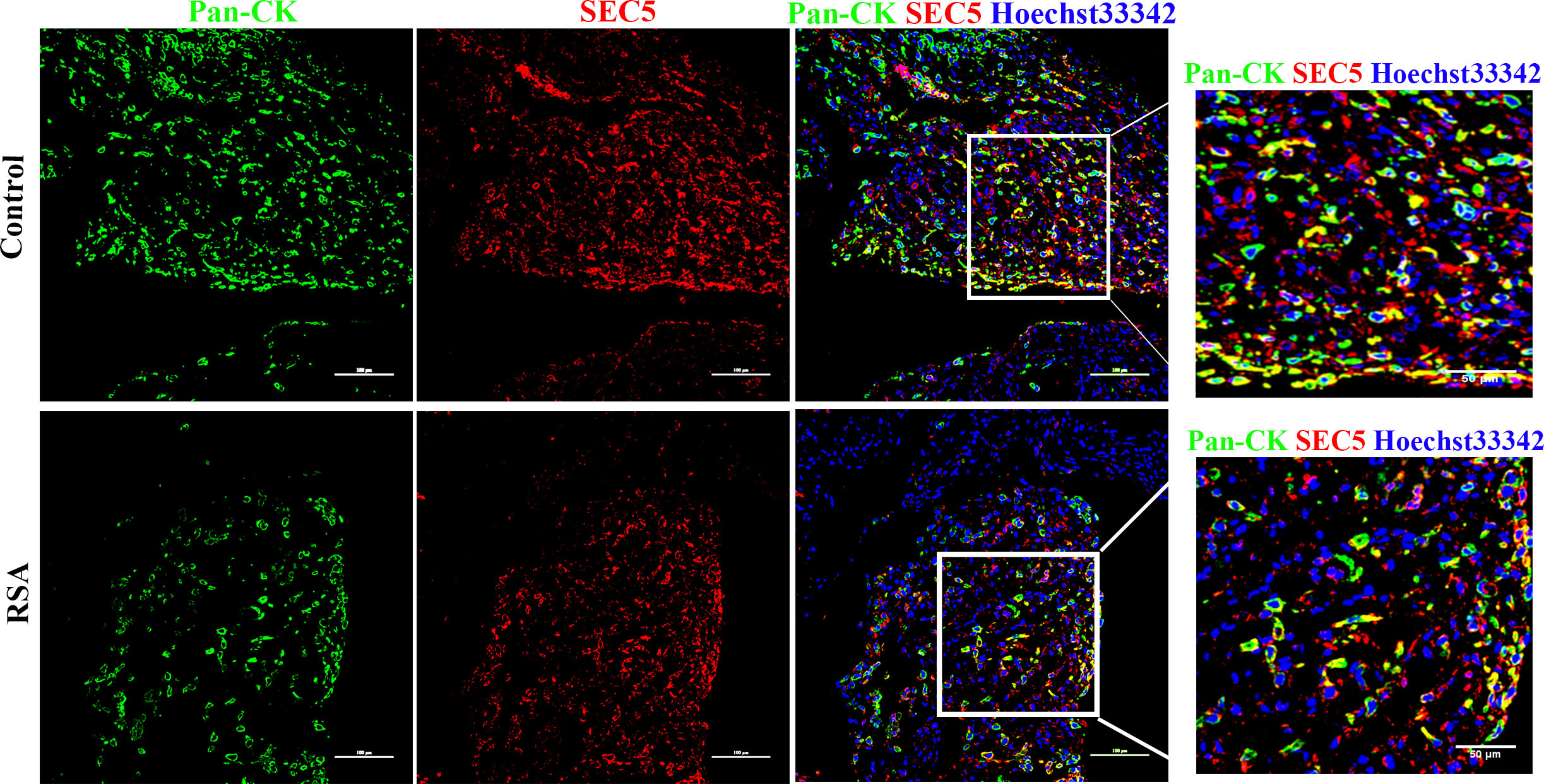

Supplement: Supplementary file 3 [file Image1.JPEG]

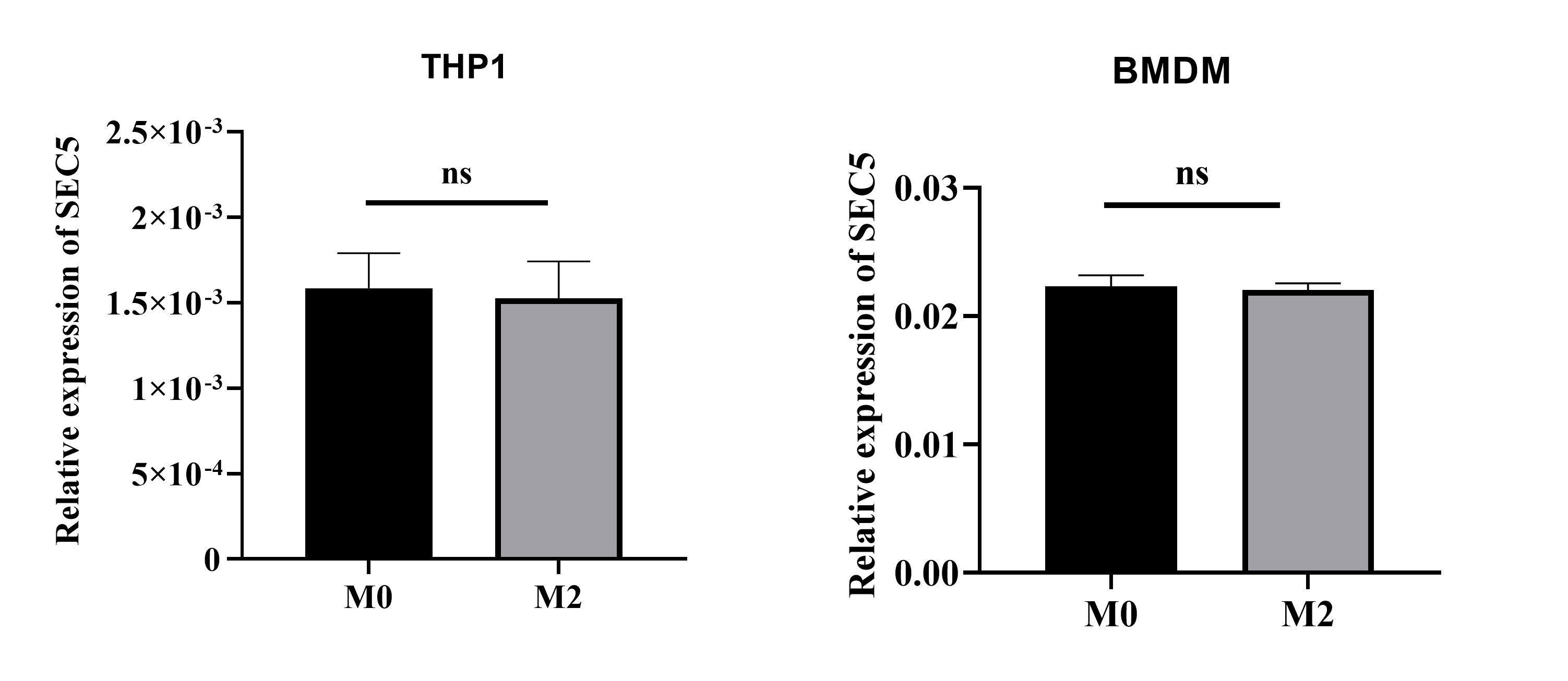

Supplement: Supplementary file 4 [file Image4.JPEG]

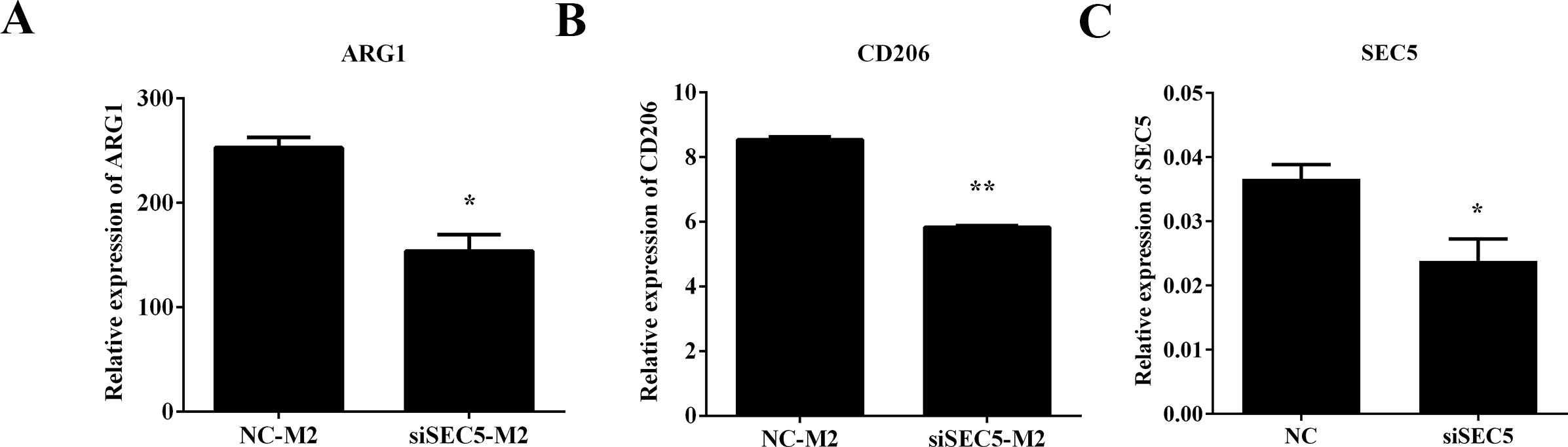

Supplement: Supplementary file 5 [file Image2.JPEG]

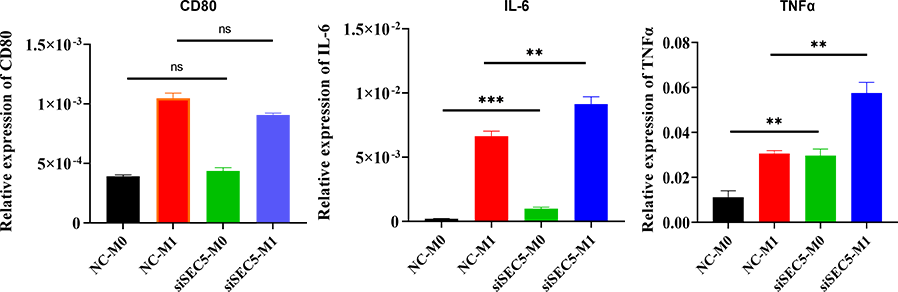

Supplement: Supplementary file 9 [file Image5.TIF]

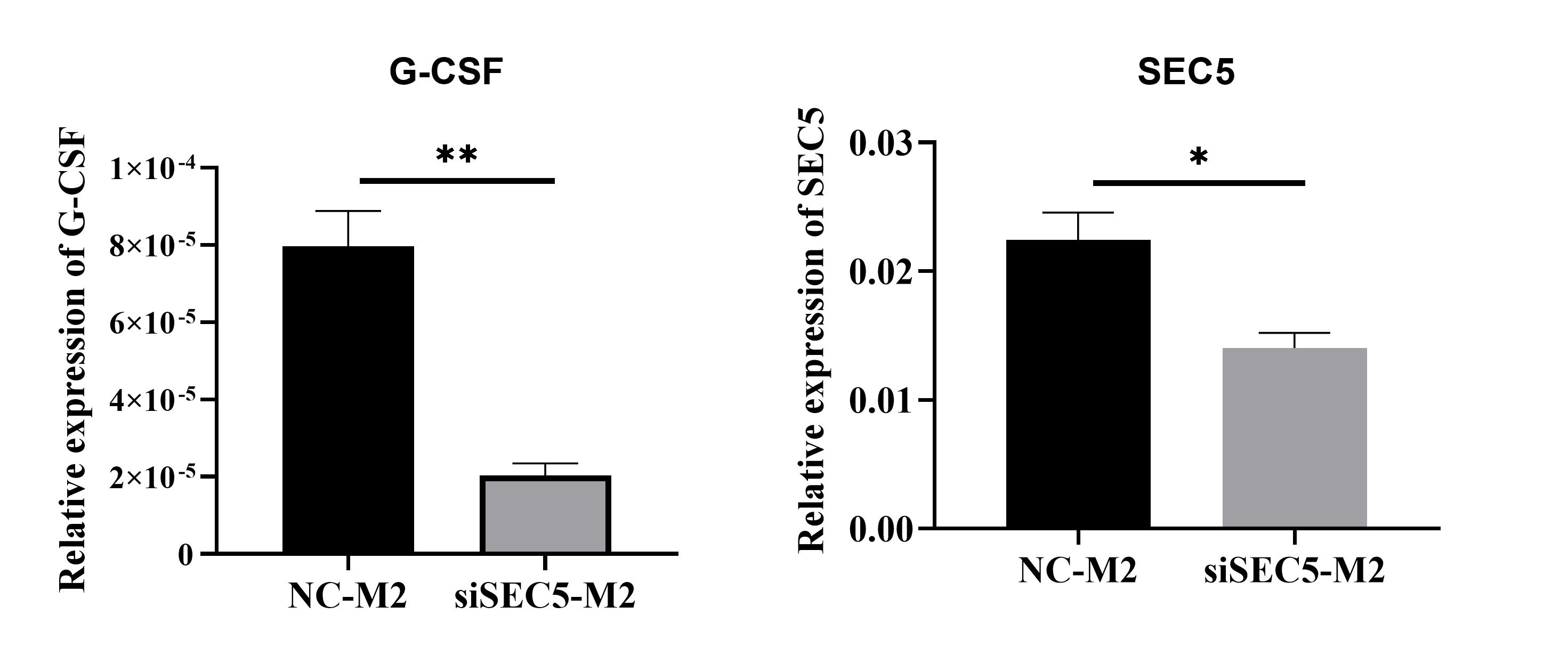

Supplement: Supplementary file 10 [file Image6.JPEG]
