# Supplementary material for: SEC5 is involved in M2 polarization of macrophages via the STAT6 pathway, and its dysfunction in decidual macrophages is associated with recurrent spontaneous abortion
Source: Front Cell Dev Biol. 2022 Oct 14;10:891748. doi: 10.3389/fcell.2022.891748 (PMC9614079; doi:10.3389/fcell.2022.891748)
Supplement: Supplementary file 2 [file Table1.DOCX]

**Table S1.** Clinical characteristic of recurrent spontaneous abortion (RSA) patients and normal pregnant (Control) women whose decidua tissues were used in this study

| **Group** | **n** | **Age**  **(years)** | **Gestational Week** | **Pregnant history** | **Childbearing history** | **Miscarriage history** |
| --- | --- | --- | --- | --- | --- | --- |
| Control | 16 | 33± 4.9 | 7.8 ± 0.8 | 0.7 ± 0.6 | 0.6 ± 0.6 | 0.0 ± 0.0 |
| RSA | 16 | 32.0 ± 5.0 | 7.9 ± 0.6 | 2.5 ± 0.6 | 0.2 ± 0.4 | 2.3 ± 0.6 |
| *P* value | |  |  | <0.001 |  | <0.001 |
