# Supplementary material for: SEC5 is involved in M2 polarization of macrophages via the STAT6 pathway, and its dysfunction in decidual macrophages is associated with recurrent spontaneous abortion
Source: Front Cell Dev Biol. 2022 Oct 14;10:891748. doi: 10.3389/fcell.2022.891748 (PMC9614079; doi:10.3389/fcell.2022.891748)
Supplement: Supplementary file 6 [file Table2.DOCX]

**Table S2.** Primers used for real-time PCR

| **Primer name** | **Sequence (5′to3′)** |
| --- | --- |
| Homo SEC5 Forward | GCTGTGTGGGTTGATGAAATGA |
| Homo SEC5 Reverse | GTTAGCAGGACGTAAGGACAAG |
| Homo actin Forward | CATGTACGTTGCTATCCAGGC |
| Homo actin Reverse | CTCCTTAATGTCACGCACGAT |
| Homo CD206 Forward | CTGAATTGTACTGGTCTGTCCT |
| Homo CD206 Reverse | GCTTAGATGTGGTGCTGTGG |
| Homo CCL22 Forward | ATTACGTCCGTTACCGTCTGC |
| Homo CCL22 Reverse | TCCCTGAAGGTTAGCAACACC |
| Homo TGFβ Forward | CTAATGGTGGAAACCCACAACG |
| Homo TGFβ Reverse | TATCGCCAGGAATTGTTGCTG |
| Mus SEC5 Forward | GGGAAAACGGAGGTGCAAGT |
| Mus SEC5 Reverse | CTGGTCATGTAAGGTTGATGGAG |
| Mus ARG1 Forward | CTCCAAGCCAAAGTCCTTAGAG |
| Mus ARG1 Reverse | AGGAGCTGTCATTAGGGACATC |
| Mus CD206 Forward | CTCTGTTCAGCTATTGGACGC |
| Mus CD206 Reverse | CGGAATTTCTGGGATTCAGCTTC |
| Mus TNFα Forward | CCCTCACACTCAGATCATCTTCT |
| Mus TNFα Reverse | GCTACGACGTGGGCTACAG |
| Mus IL6 Forward | TAGTCCTTCCTACCCCAATTTCC |
| Mus IL6 Reverse | TTGGTCCTTAGCCACTCCTTC |
| Mus GAPDH Forward | ACCCAGAAGACTGTGGATGG |
| Mus GAPDH Reverse | TTCAGCTCAGGGATGACCTT |
