# Supplementary material for: SEC5 is involved in M2 polarization of macrophages via the STAT6 pathway, and its dysfunction in decidual macrophages is associated with recurrent spontaneous abortion
Source: Front Cell Dev Biol. 2022 Oct 14;10:891748. doi: 10.3389/fcell.2022.891748 (PMC9614079; doi:10.3389/fcell.2022.891748)
Supplement: Supplementary file 7 [file Table3.DOCX]

**Table S3.** Information of antibodies used in this study

| **Antibodies** | **Catalog Number** | **Company** |
| --- | --- | --- |
| Anti-EXOC2 (SEC5) | HPA032093 | Sigma-Aldrich |
| EXOC2 (SEC5) Rabbit pAb | A19948 | ABclonal |
| Anti-Mannose Receptor (CD206) antibody | ab8918 | abcam |
| Normal Rabbit IgG | #2729 | Cell Signaling Technology |
| Anti-STAT6 antibody | ab32520 | abcam |
| Anti-STAT6 (phospho Y641) antibody | ab263947 | abcam |
| Pan-Keratin Mouse mAb | #4545 | Cell Signaling Technology |
| Phospho-Jak1(Tyr1034/1035) | #3331 | Cell Signaling Technology |
| Anti-JAK1 antibody | ab133666 | abcam |
| Anti-JAK3 antibody | ab45141 | abcam |
| IRDye 800CW Goat anti-Rabbit IgG (H + L) | 925-32211 | LI-COR |
| Donkey anti-rabbit (H+L) Cy3 | AP182C | EMD Millipore |
| Donkey anti-Mouse IgG (H+L) Highly Cross-Adsorbed Secondary Antibody, Alexa Fluor 488 | A-21202 | Thermo Fisher |
