# Supplementary material for: SEC5 is involved in M2 polarization of macrophages via the STAT6 pathway, and its dysfunction in decidual macrophages is associated with recurrent spontaneous abortion
Source: Front Cell Dev Biol. 2022 Oct 14;10:891748. doi: 10.3389/fcell.2022.891748 (PMC9614079; doi:10.3389/fcell.2022.891748)
Supplement: Supplementary file 8 [file Table4.DOCX]

The original files for microscopy images and flow cytometry data were shared online （https://www.jianguoyun.com/p/Dcv244kQjtOlChjB9a4E）.
